# The expression and antigenicity of a truncated spike-nucleocapsid fusion protein of severe acute respiratory syndrome-associated coronavirus

**DOI:** 10.1186/1471-2180-8-207

**Published:** 2008-11-28

**Authors:** Feng Mu, Dongsheng Niu, Jingsong Mu, Bo He, Weiguo Han, Baoxing Fan, Shengyong Huang, Yan Qiu, Bo You, Weijun Chen

**Affiliations:** 1 Beijing Institute of Genomics, Chinese Academy of Sciences, Airport industry B6, Beijing, 101300, PR China; 2 State Key Laboratory of Pathogen and Biosecurity, Institute of Microbiology and Epidemiology, Academy of Millitary Medical Sciences, Dongdajie Road 20, Beijing, 100071, PR China; 3 302 Hospital, Fengtai Road 26, Beijing 100853, PR China; 4 301 Hospital, Fuxing Road 28, Beijing 100039, PR China; 5 Beijing Red Cross Blood Center, North middle Sanhuan Road 37, Beijing 100088, PR China

## Abstract

**Background:**

In the absence of effective drugs, controlling SARS relies on the rapid identification of cases and appropriate management of the close contacts, or effective vaccines for SARS. Therefore, developing specific and sensitive laboratory tests for SARS as well as effective vaccines are necessary for national authorities.

**Results:**

Genes encoding truncated nucleocapsid (N) and spike (S) proteins of *SARSCoV *were cloned into the expression vector *pQE30 *and fusionally expressed in *Escherichia coli *M15. The fusion protein was analyzed for reactivity with SARS patients' sera and with anti-sera against the two human coronaviruses *HCoV *229E and *HCoV *OC43 by ELISA, IFA and immunoblot assays. Furthermore, to evaluate the antigen-specific humoral antibody and T-cell responses in mice, the fusion protein was injected into 6-week-old BALB/c mice and a neutralization test as well as a T-cell analysis was performed. To evaluate the antiviral efficacy of immunization, BALB/c mice were challenged intranasally with *SARSCoV *at day 33 post injection and viral loads were determined by fluorescent quantitative RT-PCR. Serological results showed that the diagnostic sensitivity and specificity of the truncated S-N fusion protein derived the SARS virus were > 99% (457/460) and 100.00% (650/650), respectively. Furthermore there was no cross-reactivity with other two human coronaviruses. High titers of antibodies to *SRASCoV *appeared in the immunized mice and the neutralization test showed that antibodies to the fusion protein could inhibit *SARSCoV*. The T cell proliferation showed that the fusion protein could induce an antigen-specific T-cell response. Fluorescent quantitative RT-PCR showed that BALB/c mice challenged intranasally with *SARSCoV *at day 33 post injection were completely protected from virus replication.

**Conclusion:**

The truncated S-N fusion protein is a suitable immunodiagnostic antigen and vaccine candidate.

## Background

The epidemic of severe atypical pneumonia, designated "severe acute respiratory syndrome (SARS)" by the World Health Organization (WHO) and first observed in Guangdong Province of China in November 2002, affected 8422 people and caused 916 deaths in 33 countries and areas worldwide up to August 7, 2003 [1,2]. A novel coronavirus, SARS-associated coronavirus (*SARSCoV*), was confirmed as the pathogen [3-6]. In the absence of effective drugs, controlling this disease relies on the rapid identification of cases and appropriate management of the close contacts, or effective vaccines against SARS. Therefore, the development of both specific and sensitive laboratory tests for SARS as well as effective vaccines is necessary for national authorities.

Laboratory tests for SARS based on indirect immunofluorescence assay (IFA) or viral particle lysate enzyme-linked immunosorbent assay (*SARSCoV *lysate ELISA) to detect antibodies against *SARSCoV *are important methods [7]. However, these methods both require cultivation of *SARSCoV *in a biosafety level 3 or 4 laboratory, which is both dangerous and difficult. Finding a suitable diagnostic test for this virus therefore remains a high priority. A practical approach towards this goal is to clone and express the immunodominant genes of *SARSCoV*.

Several studies have shown that most of the antigenic epitopes of *SARSCoV *are located on the nucleocapsid (N) and spike (S) proteins and that the latter protein has an important role in viral entry and pathogenesis [8-12]. Other data have shown that the viral N and S proteins of coronaviruses could induce a specific T cell response [13-16]. Here, we report the cloning and expression of a truncated S-N fusion protein of *SARSCoV *and the investigation of its antigenicity and immunogenicity.

## Methods

### Viruses and vectors

The *pQE30 *vector was purchased from Qiagen (Qiagen GmbH, Hilden, Germany). *Escherichia coli *M15 was used as host strain for *the *vector. The following virus strains were kindly provided by the Academy of Military Medical Science and the National Institute for the Control of Pharmaceutical and Biological Products: The *SARSCoV *(BJ01); *SARSCoV *(GD01); human coronavirus 229E (*HCoV*229E) and human coronavirus OC43 (*HCoV*OC43). All work with infectious virus was performed in a biosafety level 3 laboratory.

### Construction of recombinant expression plasmids

Viral RNA was extracted with TRIzol according to manual (Invitrogen). All primers were synthesized by the Shanghai Sangon Company according to the published DNA sequences (table 1). Genomic *SARSCoV *sequences for N protein as well as for truncated N (321-422aa) and S (264-680aa) proteins were amplified by RT-PCR in a mixture of 200 μM (each) deoxynucleoside triphosphate, 0.3 μM (each) primer, 1 U of *Taq *polymerase (Takara) in 10 mM Tris-HCl buffer (pH 8.3) supplemented with 2.0 mM MgCl_2 _and 50 mM KCl. The PCR reactions were started with 10 min at 95°C and followed by 35 cycles, with 1 cycle consisting of 45 sec at 94°C, 30 sec at 55°C, and 60 sec at 72°C. A final step of 5 min at 72°C was added to the last cycle. The fusion gene construct was established for expression of a truncated S-N fusion protein. The recombinant plasmids were constructed as described elsewhere [17]. All restriction enzymes and ligases were purchased from TaKaRa biotechnology Co., Ltd (Dalian, China). *E. coli *M15 was transformed with ligation mixtures and the control vector, respectively. The presence of the target genes in the recombinant plasmids was verified by gene-specific PCR and sequence analysis.

**Table 1:** Primers used for target gene by RT-PCR

Primer^a^	Primer sequences^b^	PCR product (bp)
SARS-Nf-4	5'- cgc *ggatcc*tct gat aat gga ccc ca -3'	1266
SARS-Nr-1269	5'- gc *ctgcag*tta tgc ctg agt tga atc agc aga -3'	
229E-Nf-4	5'- cgc *ggatcc*gct aca gtc aaa tgg gct gat -3'	1167
229E-Nr-1170	5'- ccc *gtcgac*tta gtt tac ttc atc aat tat -3'	
OC43-Nf-4	5'- cgc *ggatcc*tct ttt act cct ggt aag caa -3'	1344
OC43-Nr-1347	5'- ccc *aagctt*tta tat ttc tga ggt gtc ttc -3'	
SARS-tNf-961	5'- cgc *ggtacc*att ggc atg gaa gtc aca -3'	309
SARS-tNr-1269	5'- ccc *ctgcag*tta tgc ctg agt tga atc agc aga -3'	
229E-tNf-925	5'- cgc *ggatcc*gtt tcc aaa gag tca ggc aac -3'	246
229E-tNr-1170	5'- ccc *gtcgac*tta gtt tac ttc atc aat tat -3'	
OC43-tNf-985	5'- cgc *ggatcc*tta gag ttg gcc aaa gtg -3'	363
OC43-tNr-1347	5'- ccc *aagctt*tta tat ttc tga ggt gtc ttc -3'	
SARS-tSf-790	5'- cgc *ggatcc*ctc aag tat gat gaa aat ggt aca atc aca -3'	1251
SARS-tSr-2040	5'- gc *ggtacc*aga cat agt ata agc cac aat aga -3'	

^a ^f and r signify forward and reverse primers, respectively.

^b ^underlined sequences indicate restriction sites: *Bam*HI (ggatcc), *Pst*I (ctgcag), *Sal*I (gtcgac), *Hin*dIII (aagctt), and *Kpn*I (ggtacc).

### Expression and purification of the recombinant proteins

**Figure 1:**
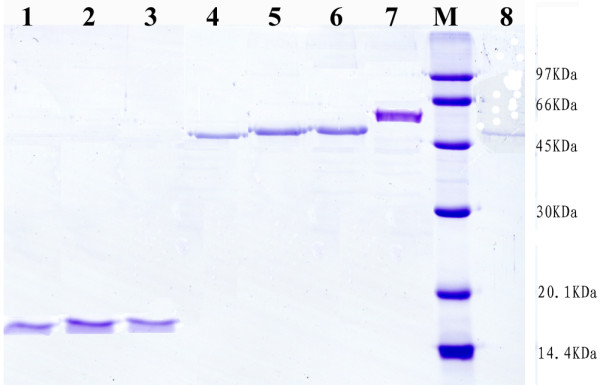
**SDS-PAGE analysis of the expression and purification target proteins**. Lane 1: purified *HCoV*229E truncated N protein; Lane 2: purified *SARSCoV *truncated N protein; Lane 3: purified *HCoV*OC43 truncated N protein; Lane 4: purified *HCoV*229E N protein; Lane 5: purified *SARSCoV *N protein; Lane 6: purified *HCoV*OC43 N protein; Lane 7: purified *SARSCoV *truncated S-N fusion protein; Lane 8: purified *SARSCoV *truncated S protein; Lane M: protein molecular weight marker.

The materials and methods used for obtaining the recombinant proteins were described in detail elsewhere [17]. The transformed bacteria were induced with 2.0 mM IPTG at 37°C and inclusion bodies containing recombinant proteins with N-terminal sequences of six consecutive His residues were serially extracted with 2 M urea and then dissolved in 8 M urea. It was then subjected to purification by means of a Ni-NTA Affinity Chromatography Purification Kit according to manual (Qiagen GmbH). Recovery of purified and renatured recombinant proteins from the denatured state in 8 M urea in buffer A (10 mM Tris-Cl (pH 7.0), 100 mM NaH_2_PO_4_) was achieved by sequential dialysis against 6 M, 4 M and 2 M urea in buffer A and finally against buffer A only. In detail, the products were dialyzed twice against 5 volumes of 6 M urea in buffer A for 30 min at room temperature. The same procedure was repeated with 4 M urea and 2 M urea in buffer A. The final dialysis was against buffer A with two initial changes of buffer after 30 min each and a final dialysis overnight at 4°C. The purity of the target proteins was determined by SDS-PAGE [18], see Figure 1.

#### Human sera

SARS patients' sera: 460 serum samples from SARS convalescents (from 35 to 114 days after the onset of illness) fulfilling the clinical WHO case definition of SARS, and whose diagnosis was subsequently confirmed by seroconversion, were collected in 301 Hospital (Beijing, China) and 302 Hospital (Beijing, China). All sera were tested positive by the *SARSCoV *lysate ELISA IgG Kit (Beijing BGI-GBI Biotech Corp.), which has been approved by the State Food and Drug Administration (SFDA) for the detection of anti-*SARSCoV *immunoglobulin (Ig) G antibody from human serum or plasma specimens.

#### Control sera

Sera from 650 Healthy blood donors were collected by the Beijing Red Cross blood center from May to October 2003.

#### Mouse Sera

Polyclonal mouse sera against *SARSCoV *(BJ01), *HCoV *229E and *HCoV *OC43 were prepared in our laboratory (IFA dilution: 1:5120, 1:5120, 1:10240, respectively) as were polyclonal mouse sera against purified recombinant proteins *SARSCoV *N, *HCoV *OC43 N, *HCoV *229E N (IFA dilution: 1:5120, 1:2560, 1:5120, respectively). Control sera were collected from healthy BALB/c mice.

### Immunization

Mouse immunization was performed according to established protocols [19]. Briefly, sixteen 6-week-old BALB/c mice were divided into 2 groups and injected subcutaneously with 0.1 mL of purified recombinant fusion truncated S-N protein solution (100 μg/mL) and PBS, respectively, both mixed with an equal volume of paraffin oil. Immunized mice were boosted after 24 days using the half dose of antigen by celiac arterial route.

### Immunological analyses

#### Immunoblot analysis

An immunoblot analysis was performed as described in detail elsewhere [18]. SDS-PAGE analysis was performed using the Mini-protein 3 Electrophoresis System (BIO-RAD). The stacking gel and separation gel contained 5% and 15% acrylamide, respectively. Electrophoresis was carried out at a constant voltage of 120 V for 180 min. The proteins were electroblotted onto nitrocellulose membranes. The mouse sera were then tested against each of the recombinant proteins.

#### IFA

An indirect immunofluorescence assay (IFA) was performed to detect antibodies to *SARSCoV *(BJ01), *HCoV *229E and *HCoV *OC43 by using *SARSCoV *according to established protocols [20].

#### ELISA

Microtiter plates (96 wells, Shenzhen Jinchanhua Co. Ltd) were coated overnight at 4°C with either of the eight recombinant antigens (four recombinant antigens of *SARSCoV*: truncated S protein, N protein, truncated N protein, and truncated S-N fusion protein; two recombinant antigens of *HCoV*229E: truncated N protein, N protein; two recombinant antigens of *HCoV*OC43: truncated N protein, N protein) diluted in 50 mM NaHCO_3 _buffer (pH 9.6). Each well was rinsed with PBS (phosphate-buffered saline) containing 0.05% Tween-20 and 3% BSA for blocking the remaining protein-binding sites. After incubation at 37°C for 1 hour, the plates were washed five times with the PBS/Tween-20 buffer. Diluted serum samples (1:10 with PBS) were added to the plates. The plates were incubated at 37°C for 30 min and washed five times with the PBS/Tween-20 buffer. After addition of peroxidase-conjugated goat anti-human IgG (diluted 1:2000 in PBS supplemented with 0.5% of Tween-20 and 1.5% of BSA) to each well and the plates were incubated at 37°C for 30 min, then washed five times with the PBS/Tween-20 buffer before the addition of tetramethyl-benzidine (TMB)/hydrogen peroxide substrate. Reaction was stopped by addition of 2 M H_2_SO_4_. The OD_450/630 _value was measured with a microtiter plate reader in triplicates. A blank control, a negative control and a positive control were always included on each plate. The cut-off values for IgG were 0.16 (three N proteins), 0.12 (truncated S protein), 0.14 (three truncated N proteins), 0.12 (truncated S-N protein), respectively, which were calculated as the mean + 2 SD of the readings given by 1000 blood donor control sera collected from 2001 to 2002 in Beijing. Samples were tested again in triplicates when their OD_450/630 _values were near the cut-off values. For the detection of mice antibodies, all procedures were the same as for detection of human antibodies except that peroxidase-conjugated goat anti-mice IgG diluted to 1:1000 was used. Mice sera were diluted to 1:20 with PBS.

#### Neutralization test

Neutralizing titer (NT) of mouse sera was measured by a rapid microneutralization assay [21]. In brief, heat-inactivated (55°C for 30 min) mouse immune serum was diluted tenfold and then serially diluted twofold to 1:2560 in DMEM (Gibco) containing 5% heat-inactivated fetal calf serum (56°C for 30 min). Approximately 50 μL of *SARSCoV *(BJ01 strain) (400 TCID_50_/100 μL) was mixed with an equal volume of diluted serum and incubated at 35°C for 1 h; then 50 uL of the mixture (containing 100 TCID_50_) and 50 μL of DMEM containing 5% inactivated fetal calf serum were added onto a VeroE6 cell monolayer in triplicate. The viral cytopathic effect (CPE) was observed on days 2 and 3. The dilution of serum that completely prevented CPE in 50% of the wells was calculated according to the Reed Muench formula [22].

#### Cross-reactivity among viruses and recombinant proteins

Serological cross-reactivity among different human coronaviruses was tested by incubation of *SARSCoV*-infected cells with mouse antisera against the two other human coronaviruses, *HCoV*229E and *HCoV*OC43, and subsequent indirect immunofluorescence assay (IFA). To evaluate cross-reactivity among different recombinant proteins of the three human coronaviruses, the proteins were subjected to immunoblot assays with mouse antisera against the proteins as well as antisera against the viruses and were also subjected to ELISA with 460 serum samples from SARS convalescents as well as mice antisera against the viruses.

### Immune responses to the truncated *SARSCoV *S-N fusion protein

#### Humoral immune response

Serum samples were collected from the tail veins every 3 days after the initial immunization and the final serum samples were collected from the orbital plexus for antibody level assessment by the *SARSCoV *lysate ELISA IgG Kit according to the manufacturer's instruction, except that peroxidase-conjugated goat anti-human IgG was substituted by peroxidase-conjugated goat anti-mouse IgG (Sihuan Sci-Technics Company, Beijing). A value of S/N ≥ 2.1 was taken as positive standard.

#### Spleen lymphocyte immune response

The proliferation of spleen lymphocytes was measured by colorimetric analysis described previously [23]. Four BALB/c mice in each group were killed on day 33 after immunization and their spleens were ground into single-cell suspensions in RPMI 1640 medium (Gibco) supplemented with 10% fetal calf serum. The suspensions were mixed with 5 volumes of erythrocyte lysis buffer (0.01 M Tris-HCl pH 7.6; 0.01 M NaCl; 0.005 M MgCl_2_), incubated for 10 minutes on ice and centrifuged at 400 g for 5 min at 4°C. The pellets were resuspended in RPMI 1640 medium (Gibco) supplemented with 10% fetal calf serum. They were seeded in triplicates in flat-bottom 96-well microtiter plates (Costar) with 5 × 10^5 ^cells per well in 100 μL of culture medium with purified and truncated S-N protein at 10, 3, 1, 0.3 and 0 μg/mL, respectively. After incubation for 3 days with 5% CO_2 _at 37°C, 10 μL of a solution of the tetrazolium salt 3-(4,5-dimethylthiazol-2-yl)-2,5-diphenyltetrazolium bromide (MTT) was added to each well, and the plates were incubated for 4 h at 37°C. One hundred microliter of lysis buffer containing 10% Triton-50% isopropanol-0.01 M hydrochloric acid was then added to each well, and the plates were incubated overnight. The optical densities at 570 nm (OD_570_) and at 630 nm (OD_630_) were measured.

### *SARSCoV *challenge and determination of virus load

Four BALB/c mice from each group, immunized as well as controls, were challenged intranasally with 10^4 ^TCID_50 _of the *SARSCoV *GD01 strain on day 33 after immunization to tested the heterologous protection. After two days of clinical observation the mice were sacrificed and their lungs were collected for determination of the level of viral RNA.

Fifty mg lung tissue was first homogenized in liquid nitrogen and then in 1 mL of TRIzol. Tissue homogenates were clarified by low-speed centrifugation (3000 rpm). RNA extraction was performed according to manual (Invitrogen). Virus loads were determined by fluorescent quantitative RT-PCR and expressed as number of copies per gram tissue [24].

## Results

### Serological cross-reactivity among coronaviruses and recombinant proteins

**Figure 2:**
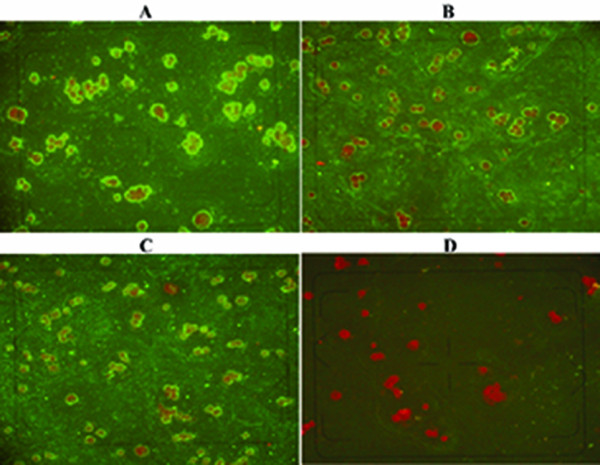
**IFA analysis of cross-reaction of the *SARSCoV *infected cell using mouse anti-sera to recombinant N proteins of other two human Coronaviruses**. A: Reacted with mouse anti-serum to *SARSCoV *N protein; B: Reacted with mouse anti-serum to *HCoV*229E N protein; C: Reacted with mouse anti-serum to *HCoV*OC43 N protein; D: Reacted with mouse serum from controls injected with PBS;

**Figure 3:**
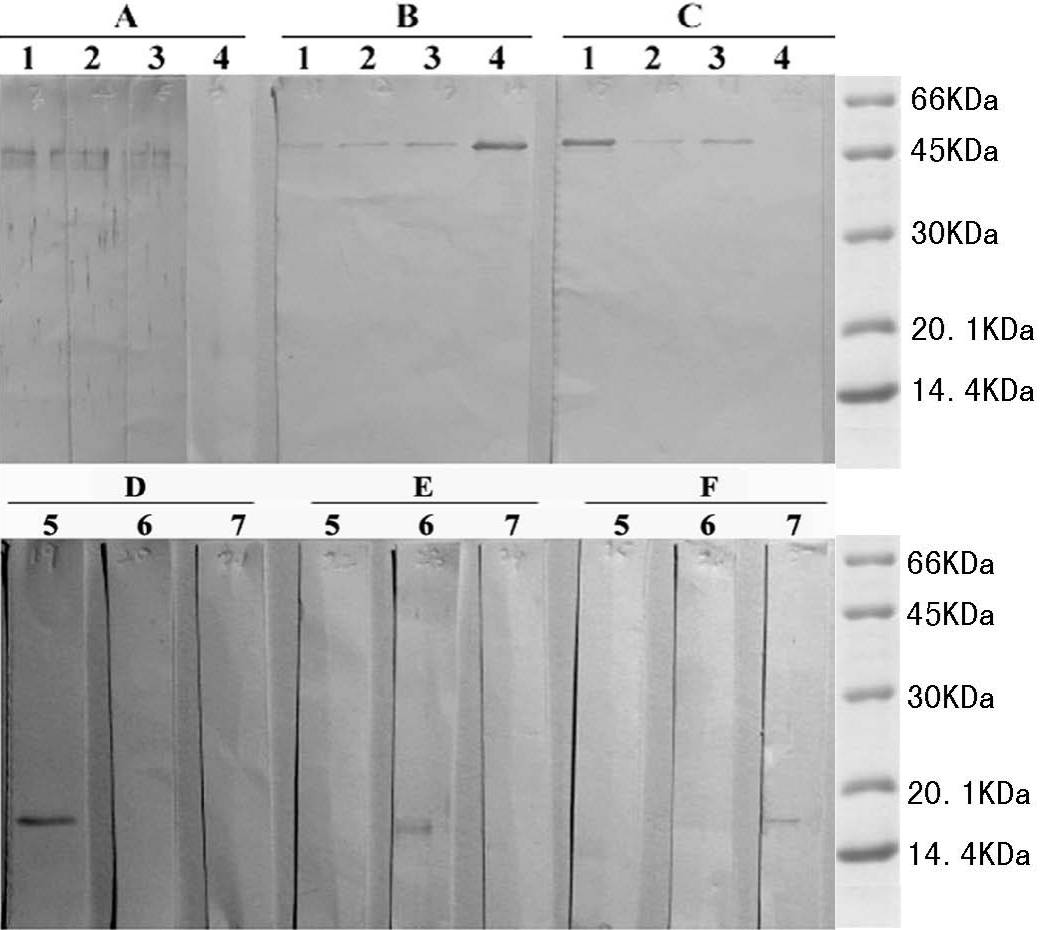
**Immunoblot assay analysis of cross-reaction of the coronaviruses recombinant proteins using mouse anti-sera to three different human Coronaviruses and recombinant proteins**. A: Reacted with mouse anti-serum to *HCoV229E*; B: Reacted with mouse anti-serum to *SARSCoV*; C: Reacted with mouse anti-serum to *HCoVOC43*; Lane 1: Purified *HCoV229E *N protein; Lane 2: Purified *HCoVOC43 *N protein; Lane 3: Purified *SARSCoV *N protein; Lane 4: Purified *SARSCoV *truncated S protein; D: Reacted with mouse anti-serum to *SARSCoV*; E: Reacted with mouse anti-serum to *HCoV229E*; F: Reacted with mouse anti-serum to *HCoVOC43*; Lane 5: Purified *SARSCoV *truncated N protein; Lane 6: Purified *HCoV229E *truncated N protein; Lane 7: Purified *HCoVOC43 *truncated N protein.

The result of immunofluorescence analysis of cross-reactivity between *SARSCoV*-infected cells and mouse antisera against each N protein of the three coronaviruses is shown in Figure 2. The results show that mouse anti-sera to *HCoV*229E N protein and *HCoV*OC43 N protein cross-react with *SARSCoV*. Figure 3 shows the result of immunoblot analyses for cross-reactions between (i) intact N protein from the three human coronaviruses plus the truncated S protein and mouse antisera against each coronavirus (panels A, B, and C) and (ii) truncated N protein from each coronavirus and mouse antisera against each coronavirus (panels D, E and F). The results showed (a) cross-reactions between the intact N proteins and the antisera against the three coronaviruses and (b) no cross-reaction between the antisera and the truncated proteins. The recombinant proteins of *HCoV*229E and *HCoV*OC43 were also tested with 460 serum samples from SARS convalescents by ELISA. The results showed 34 and 21 sera were tested positive by N proteins of *HCoV*OC43 and *HCoV*229E respectively, while none of the sera were tested positive by both truncated N proteins of *HCoV*OC43 and *HCoV*229E.

### Diagnostic sensitivity and specificity of ELISA with truncated S-N fusion protein as antigen

The qualities of truncated S protein, N protein, truncated N protein and truncated S-N fusion protein as diagnostic antigens were evaluated by ELISA technique as described in Materials and Methods. All 460 SARS patients' sera were tested against all four antigens. Only 3 samples were tested negative in the truncated S-N fusion protein assay, whereas 62, 38 and 43 samples were tested negative in the truncated S protein, N protein and truncated N protein assays, respectively (Table 2). Compared with *SARSCoV *lysate ELISA, the sensitivity of ELISA with truncated S protein, N protein, truncated N protein and truncated S-N fusion protein as antigens were 86.5% (398/460), 91.7% (422/460), 90.7% (417/460) and > 99% (457/460), respectively. To evaluate the specificity of these assays, the sera of the 650 healthy people were tested. Only a few gave a positive reaction with either *SARSCoV *lysate or N protein, whereas none reacted against the truncated proteins (Table 2). To further evaluate the specificity of the truncated S-N fusion protein, the polyclonal mouse sera against *SARSCoV *(BJ01), *HCoV*229E and *HCoV*OC43 antisera to were tested by ELISA. Only mice antiserum against *SARSCoV*(BJ01) tests positive.

**Table 2:** Antibody detection rates for recombinant protein ELISA and *SARSCoV *lysate ELISA of sera from SARS patients and healthy controls.

Coating antigen	Sera of SARS patients		Sera of healthy blood donors	
		
	Positive	negative	Positive	negative
*SARSCoV *lysate	460	0	5	645
truncated S protein	398	62	0	650
N protein	422	38	8	642
truncated N protein	417	43	0	650
truncated S-N protein	457	3	0	650

### Specific humoral and cellular anti- *SARSCoV *immune responses to the truncated S-N fusion protein

Sera antibody tests showed the ability of the fusion protein to induce the generation of SARS-specific antibodies in the immunized mice. Nine to twelve days after injection, the specific Ig G antibody could be detected. To test whether the mouse sera against the truncated S-N protein were able to neutralize *SARSCoV*, a property that is likely to be crucial in the defense against virus infection, the *SARSCoV *BJ01 strain was used in a microneutralization assay as described in Materials and Methods. The serum titer of neutralizing antibodies against *SARSCoV *was 2.425 ± 0.209 (Lg dilution ± SD)

The lymphocytes proliferation assay showed that the truncated S-N protein could induce T cell proliferation of mice immunized with the truncated fusion protein (Table 3). There are significant differences between mice immunized with the truncated fusion protein and the controls (t test *p *= 0.0084 < 0.05).

**Table 3:** Effects of recombinant protein on spleen cell proliferation in mice injected with recombinant proteins and a control group of mice injected with PBS by MTT^a^

Group	Proliferation (D value^b ^± SD) with the following concentration (μg/ml) of recombinant proteins				
	
	10	3	1	0.3	0
PBS Control	0.116 ± 0.112	0.074 ± 0.046	0.011 ± 0.002	0.022 ± 0.008	0.043 ± 0.008
truncated S-N	0.603 ± 0.118	0.551 ± 0.019	0.542 ± 0.216	0.476 ± 0.274	0.068 ± 0.021

^a ^Four BALB/c mice of each group were killed. Spleens were harvested and lymphocyte cultures (Three repeats) were stimulated in vitro for 3 days with medium or with various concentrations of purified recombinant fusion truncated S-N protein (three repeats). The truncated S-N protein could induce T cell proliferation and significant differences were found between the groups of mice immunized with recombinant protein or injected with PBS (T test P = 0.0084 < 0.05).

^b ^D Value = D_570_-D_630_-blank

### Protection from *SARSCoV *challenge and virus replication

No clinical signs of illness were observed in either group of SARS-challenged mice. The analysis for *SARSCoV *genome copies in the lungs of immunized mice and controls was performed. The mean of virus genome copy numbers are 20708 ± 6202 (copies/g ± SD) per 1 gram lung tissue in the control group, whereas virus loads in mice immunized with truncated S-N protein were below the limit of detection.

## Discussion

SARS, a newly emerged infectious disease which caused worldwide outbreak in 2003, has been a crucial public health problem. Establishing specific and convenient laboratory tests for SARS and finding a vaccine for this virus are of high priority.

**Figure 4:**
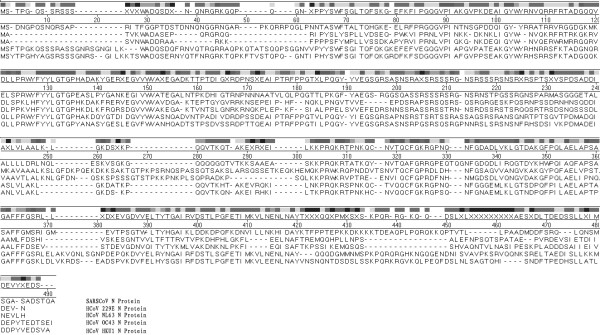
Sequence alignments of N proteins of *SARSCoV*, *HCoV*229E, *HCoV*NL63, *HCoV*OC43 and *HCoV*HKU1 by Clustal V Method with MegAlign.

Previous data have shown a high degree of sequence similarity between the nucleocapsid (N) proteins of coronaviruses and demonstrated serious serological cross-reactions [25,26]. We aligned the N protein of five human coronaviruses (*SARSCoV*, *HCoV*229E, *HCoV*NL63, *HCoV*OC43 and *HCoV*HKU1) and found several fairly homologous regions, e.g. *SARSCoV *57-210aa, 258-320aa. In these regions, the N protein of *SARSCoV *is 35–39% identical in amino acid sequence to the N protein of *HCoV *229E and *HCoV *NL63 and 47–50% identical in amino acid sequence to that of *HCoV*OC43 and *HCoV*HKU1 (Figure 4). We also found that the C-terminal of the N protein (*SARSCoV *321-422aa) has lower identity in amino acid sequence among these human coronaviruse (Figure 4). In addition, our previous research showed that some unique highly antigenic sites are located in the C-terminal part of the SARS N protein and in the 270-667aa of the SARS spike (S) protein [8]. Other research also showed that the C-terminal part of the SARS N protein was highly antigenic [27]. In the present study, using the *pQE30 *expression vector we cloned the N genes, gene segments encoding the C-terminal parts of the N proteins from all three coronaviruses, as well as *SARSCoV *gene segments encoding a truncated spike protein (264-680 aa) and a truncated S-N fusion protein, respectively. All proteins were highly expressed in *E. coli *M15. To evaluate the cross-reactivity of these recombinant proteins and viruses, IFA and immunoblot assays were performed. The results (Figures 2 and 3) showed that the truncated proteins only reacted with species-specific antiserum while the N proteins cross-reacted as did the viruses. The further ELISA results also showed that the intact N proteins of *HCoV*229E and *HCoV*OC43 cross-reacted with SARS patients' sera (21/460, 34/460 respectively) while the truncated N proteins did not cross-react with SARS patients' sera.

Using the purified proteins as antigens in ELISA assays for antibodies in the sera of SARS patients we found that the assay using truncated S-N fusion protein has a clearly higher sensitivity than those using intact N protein or truncated S and N proteins, and virtually as high as the assay using whole *SARSCoV *lysate (Table 2). The results indicated the N and S protein were complementary in detecting SARS-specific antibodies. This is consistent with previous studies [28,29]. Five positive sera to *SARSCoV *lysate antigen were all tested positive against *SARSCoV *N protein but negative against *SARSCoV *truncated N-S protein. These sera were also tested positive against N proteins of *HCoV*229E and *HCoV*OC43 (data not shown), which could be reasonably explained partly by existence of other *HCoV *infections in these humans. The truncated S-N fusion protein was also subjected to ELISA with mice antisera against *SARSCoV *(BJ01), *HCoV*229E and *HCoV*OC43. Only mice antiserum against *SARSCoV*(BJ01) tests positive. These results showed that the *SARSCoV *truncated N-S protein had high specificity. Considering the difficulty of *SARSCoV *lysate antigen production and its false positive ratio (~0.77%, Table 2), the truncated S-N fusion protein is a suitable diagnostic antigen for detection of *SARSCoV *antibodies.

The S protein of *SARSCoV *is an important determinant of tissue tropism, as it mediates virus and cellular membrane fusion. Analysis of neutralizing epitopes showed that the receptor-binding region of the S protein plays an important role in virus infection [12,30,31]. A DNA vaccine study of *SARSCoV *has also shown that the S protein can induce protective immune responses to *SARSCoV *[32]. Moreover, some data showed that the N protein of *SARSCoV *could induce specific T-cell responses and studies of animal coronaviruses have suggested that both cellular and humoral immunity contribute to protection during persistent infection [13,16,33]. Considering that our fusion protein includes the receptor-binding region of the S protein and immunodominant T-cell epitopes of the N protein, we also investigated the role of the truncated S-N protein in anti-*SARSCoV *infection. Seven to nine days after injection of the fusion protein, the mice began to show seropositive for SARS antibodies. After the first booster, all mice generated high titer of SARS-specific antibodies and the antibodies could neutralize the *SARSCoV *infectivity. In the lymphocytes proliferation assay, the truncated S-N protein could induce T cell proliferation (Table 3). Compared to the control group, the mice immunized with the truncated S-N protein were protected from *SARSCoV *challenge, as indicated by a lack of detectable viral RNA. Therefore, in addition to being a valuable diagnostic antigen the truncated S-N fusion protein is a potential candidate for the development of a SARS subunit vaccine.

## Conclusion

The truncated S-N fusion protein has high sensitivity and specificity and it is a suitable diagnostic antigen for detection of *SARSCoV *antibodies. On the other hand, it could induce the mice generated high titer of SARS-specific neutralizing antibodies and T cell proliferation. The mice immunized with the truncated S-N protein were protected from *SARSCoV *challenge. It is also a potential candidate for the development of a SARS subunit vaccine.

## Competing interests

The authors declare that they have no competing interests.

## Authors' contributions

FM carried out the genes cloning and protein expression and drafted the manuscript. DSN carried out the Viruses challenge assays and partial immunoassays. JSM carried out clinical samples collection and partial immunoassays. BH, WGH, SYH and BY participated in the samples detection. BXF and YQ participated in the samples collection. WJC designed the study, perform the data analysis and wrote the manuscript. All authors read and approved the final manuscript.
